# HIT-COVID, a global database tracking public health interventions to COVID-19

**DOI:** 10.1038/s41597-020-00610-2

**Published:** 2020-08-27

**Authors:** Qulu Zheng, Forrest K. Jones, Sarah V. Leavitt, Lawson Ung, Alain B. Labrique, David H. Peters, Elizabeth C. Lee, Andrew S. Azman, HIT-COVID Collaboration, Binita Adhikari, Brian Wahl, Chloé Sarnowski, Daniel A. Antiporta, Daniel J. Erchick, Javier Perez-Saez, Joseph Ssekasanvu, Kyu Han Lee, Laura White, Natalya S. Kostandova, Neia Prata Menezes, Nicholas W. Albaugh, Nidhi Gupta, Safia S. Jiwani, Sonia T. Hegde, Swati Srivastava, Tricia Aung, Yijing Zhang, Giulia Norton, Arnav Kalra, Ashank Khaitan, Dyuti Shah, Japnoor Kaur, Keerthana Kasi, Lajjaben Patel, Lovedeep Singh Dhingra, Mudit Agarwal, Sanil Garg, Utkarsh Goel, Vikram Jeet Singh Gill, Erum Khan, Alina Patwari, Pegah Khaloo, Deepa Joshi, Emily Blagg, Emma Pence, Holly K Nelson, Jing Fan, Lauren Forbes, Meredith Schlussel, Semra Etyemez, Shanshan Song, Udit Mohan, Yi Sun, Sunyoung Jang, Nicole Frumento, Ananyaa Sivakumar, Anna-Maria Hartner, Vedika Karandikar, Ziao (Ainsley) Yan, Evan R. Beiter, Julia Song, Leia Wedlund, Miriam Singer, Rifat Sirajur Rahman, Zain Mohammad Virk, Arjan Abar, Bruce Tiu, Tyler M. Adamson, Kiran Paudel, Honghui Yao, Yinuo Wang, E Rosalie Li-Rodenborn, Ipek Ozdemir, Martha-Grace McLean, Susan Rattigan, Brooke A. Borgert, Carlos A Moreno, Nicole Elizabeth Quigley, Chengchen Li, Nimran Kaur, Catherine Gimbrone, Sarah Elizabeth Scales, Julio Cesar Zuniga-Moya, Peter Ahabwe Babigumira, Chibueze C. Igwe, H. Echo Wang, Leon Hsieh, Stuti Misra, Kelly Bruton, Danalyn Byng, Monica Miranda-Schaeubinger, Mohammad Nasir Uddin, John R. Ticehurst, Emaline Laney, Abhimanyu Bhadauria, Vidushi Gupta, Maria Clara Selles, Akash Kartik, Anmol Singh, Divya Garg, Jasmine Saini, Jyotroop Kaur, Mannat Kaur, Lena Denis, Iniobong Ekong, Renyuanouyang, Tusabe Fred, Alison Su-Hsun Liu, Molly R. Petersen, Pascal Agbadi, Ivan Segawa, Valerie Scott, Yannan Shen, Jennifer OKeeffe, Zachary Brennan, Major Singh, Ashutosh Saini, Mercy Ndukwe, Anushiya Vanajan, Jessica Minder, Eugène Lemaitre, Li Pi, Moneet Saini, Maria Cabrera-Aguas, Hur E. Zannat, Arlinda Deng, Nhat-Lan H. Nguyen, Patrick J Hinson, Laurence Buysse, Snimarjot Kaur, Chuxuan Zhang, Chhavi Saini, Daisy Y. Shu, Hamid Alemi, Prerana Shivshanker, Rohan Bir Singh, Tina B. McKay, Xia Wang, Sophia Lee, Nicolas Petersen, Frances Zielonka, Andrew Chuang, Christel Saussier, Derek A. Dutra, Elizabeth K. Conlan, Lumen Luciano Yadriel Specter, Mais Alhariri, Ramazan Karahan, Terry Yen, Yacine Bouchene, Adam Sultanov, Navdeep Singh

**Affiliations:** 1 Department of Epidemiology, Johns Hopkins Bloomberg School of Public Health, Baltimore, Maryland USA; 2 Department of International Health, Johns Hopkins Bloomberg School of Public Health, Baltimore, Maryland USA; 3 Department of Biostatistics, Boston University School of Public Health, Boston, Massachusetts USA; 4 Infectious Disease Institute and Department of Ophthalmology, Massachusetts Eye and Ear, Harvard Medical School, Boston, Massachusetts USA; 5 Abt Associates, Inc, Massachusetts, USA; 6 All India Institute of Medical Sciences, New Delhi, India; 7 B.J.Medical College, Gujarat, India; 8 Cambridge Rindge and Latin, Massachusetts, USA; 9 Cardiac Arrhythmia Service, Massachusetts General Hospital, Massachusetts, USA; 10 Johns Hopkins Bloomberg School of Public Health, Baltimore, Maryland USA; 11 Johns Hopkins University School of Medicine, Baltimore, Maryland USA; 12 Johns Hopkins University, Baltimore, Maryland USA; 13 Harvard Medical School, Boston, Massachusetts USA; 14 Center for Public Health and Human Rights, Johns Hopkins University Bloomberg School of Public Health, Baltimore, Maryland USA; 15 Central Department of Public Health, Institute of Medicine, Kathmandu, Nepal; 16 Department of Learning, Informatics, Management and Ethics, Karolinska Institute, Stockholm, Sweden; 17 Department of Applied Mathematics and Statistics, Johns Hopkins University, Baltimore, Maryland USA; 18 Department of Biochemistry and Molecular Biology, Johns Hopkins Bloomberg School of Public Health, Maryland, USA; 19 Department of Bioengineering, University of Texas at Dallas Erik Jonsson School of Engineering and Computer Science, Texas, USA; 20 University of Florida, Florida, USA; 21 Department of Biology and Emerging Pathogens Institute, University of Florida, Florida, USA; 22 Department of Biostatistics, Harvard T.H. Chan School of Public Health, Boston, Massachusetts USA; 23 Department of Community Medicine and School of Public Health, Postgraduate Institute of Medical Education and Research, Chandigarh, India; 24 Department of Epidemiology, Columbia University Mailman School of Public Health, New York, USA; 25 Department of Epidemiology, University of Michigan Ann Arbor, Michigan, USA; 26 Department of Global Health Security, Infectious Diseases Institute, Kampala, Uganda; 27 Department of Health Planning, Research and Statistics, Health and Human Services Secretariat, Federal Capital Territory Administration, Abuja, Nigeria; 28 Department of Health Policy and Management, Johns Hopkins Bloomberg School of Public Health, Baltimore, Maryland USA; 29 Department of Molecular Microbiology and Immunology, Johns Hopkins Bloomberg School of Public Health, Baltimore, Maryland USA; 30 Department of Ophthalmology, Faculty of Medical and Health Sciences, The University of Auckland, Auckland, New Zealand; 31 Department of Pathology & Molecular Medicine, McMaster University, Ontario, Canada; 32 Department of Psychosocial Research and Epidemiology, The Netherlands Cancer Institute, Amsterdam, The Netherlands; 33 Department of Radiology, Children’s Hospital of Philadelphia, Pennsylvania, USA; 34 Discipline of Anatomy & Histology, The University of Sydney, New South Wales, Australia; 35 Division of Infectious Diseases, Department of Medicine, School of Medicine, and Department of Epidemiology, Johns Hopkins Bloomberg School of Public Health, Baltimore, Maryland USA; 36 Emory University School of Medicine, Georgia, USA; 37 Engineering and Technology, SRM Institute of Science and Technology, Tamil Nadu, India; 38 Federal University of Rio de Janeiro, Rio de Janeiro, Brazil and New York University, New York, USA; 39 Government Medical College and Hospital, Chandigarh, India; 40 Government Medical College, Amritsar, Punjab India; 41 Harvard Map Collection, Harvard Library, Boston, Massachusetts USA; 42 IE University, Segovia, Spain; 43 Infectious Diseases Institute, Kampala, Uganda; 44 International Health Program, College of Medicine, National Yang Ming University, Taipei, Taiwan; 45 Department of Pathology, Johns Hopkins University School of Medicine, Baltimore, Maryland USA; 46 Department of Nursing, Kwame Nkrumah University of Science and Technology, Kumasi, Ghana; 47 Makerere University Lung Institute, Kampala, Uganda; 48 Malaria Elimination Initiative, Global Health Group, University of California San Francisco, California, USA; 49 McGill University, Quebec, Canada; 50 Médecins Sans Frontières, New York, USA; 51 Michigan State University, Michigan, USA; 52 N.C. Medical College and Hospital, Israna, Panipat, Haryana India; 53 Neonatal Intensive Care unit, Al-Hayat National Hospital, Khamis, Saudi Arabia; 54 Netherlands Interdisciplinary Demographic Institute, The Hague, Netherlands; 55 University Medical Center Groningen, Groningen, Netherlands; 56 New York University School of Medicine, New York, USA; 57 School of Engineering, EPFL, Lausanne, Switzerland; 58 School of Public Health, Imperial College London, London, UK; 59 SGRR Medical College, Dehradun, India; 60 The University of Sydney, Save Sight Institute, Discipline of Ophthalmology, Sydney Medical School, Sydney, New South Wales Australia; 61 Tufts University, Massachusetts, USA; 62 University of Maryland School of Medicine, Baltimore, Maryland USA; 63 University of Virginia College of Arts and Sciences, Virginia, USA; 64 University of Virginia, Virginia, USA; 65 UZ Ghent, Gent, East-Flanders, Belgium; 66 Vardhman Mahavir Medical College and Safdarjung Hospital, New Delhi, India; 67 Wuhan Foreign Language School, Wuhan, China; 68 Department of Ophthalmology, Massachusetts Eye and Ear, Harvard Medical School, Boston, Massachusetts USA; 69 Massachusetts Eye and Ear, Harvard Medical School, Boston, Massachusetts USA; 70 Medical Reserve Corps, Oregon, USA; 71 ZXV Group, Washington, USA

**Keywords:** Infectious diseases, Epidemiology

## Abstract

The COVID-19 pandemic has sparked unprecedented public health and social measures (PHSM) by national and local governments, including border restrictions, school closures, mandatory facemask use and stay at home orders. Quantifying the effectiveness of these interventions in reducing disease transmission is key to rational policy making in response to the current and future pandemics. In order to estimate the effectiveness of these interventions, detailed descriptions of their timelines, scale and scope are needed. The Health Intervention Tracking for COVID-19 (HIT-COVID) is a curated and standardized global database that catalogues the implementation and relaxation of COVID-19 related PHSM. With a team of over 200 volunteer contributors, we assembled policy timelines for a range of key PHSM aimed at reducing COVID-19 risk for the national and first administrative levels (e.g. provinces and states) globally, including details such as the degree of implementation and targeted populations. We continue to maintain and adapt this database to the changing COVID-19 landscape so it can serve as a resource for researchers and policymakers alike.

## Abstract (summary)

**Table:** 

Measurement(s)	Public Health • Preventive Intervention
Technology Type(s)	digital curation
Factor Type(s)	country • date
Sample Characteristic - Location	global

Machine-accessible metadata file describing the reported data: 10.6084/m9.figshare.12724058

## Background & Summary

Since the first reported cases in December 2019^1^, Coronavirus Disease 2019 (COVID-19) has become a global pandemic and a major cause of morbidity and mortality. Due to high population susceptibility and the lack of effective therapeutics and vaccines to treat or prevent this emerging disease, many healthcare systems have been overwhelmed by a global surge in cases. In an effort to limit the transmission of Severe Acute Respiratory Syndrome Coronavirus 2 (SARS-CoV-2) and mitigate its impact on public health, national and local governments worldwide have instituted a variety of public health and social measures (PHSM, often also referred to as non-pharmaceutical interventions, NPIs) in different combinations, of varying durations, and at different time points during their epidemic trajectories. This heterogeneity serves as a natural testing bed to characterize the effectiveness and impact of different interventions on SARS-CoV-2 transmission^2^. As a clearer picture of SARS-CoV-2 transmission dynamics emerges, public health experts and policy makers are now tasked with issuing informed and urgently needed recommendations for the easing of certain restrictions, many of which are associated with significant social and economic costs.

The Health Intervention Tracking for COVID-19 (HIT-COVID) project seeks to fill a critical knowledge gap through systematic collection of PHSM data at national and sub-national levels worldwide, HIT-COVID compliments other intervention tracking efforts that have tended to capture only national policies, focus primarily on middle and/ upper income countries or use less structured approaches to data collection^3–7^. We aim to provide the resolution required for robust epidemiologic study, which may in turn inform policy makers at a national, sub-national, and local level. The project, which has mobilized an international team of trained data collectors using standardized field definitions and data collection processes, has come with careful curation and internal auditing of policy dates and source documents for optimal transparency. HIT-COVID also collections information on whether missing data truly reflect an absence of policy interventions. As the current pandemic unfolds, HIT-COVID may also provide the data necessary to analyze historical trends in disease transmission, which may be important for current and future policy making, research, and education.

## Methods

We collected information on PHSM policies related to COVID-19 worldwide at the national and first administrative (e.g. provinces, states) levels, with finer geographic resolution data for specific countries, including counties in the United States of America. We focused primarily on interventions that may have direct and quantifiable impacts on disease transmission, and limited data collection to government-level policy changes. We collected information on policies using a classification system adapted from the WHO PHSM Database^7^: (1) restrictions of travel and movement, (2) social and physical distancing measures, (3) surveillance and response measures, and (4) other measures, including military and police deployment, state of emergency declarations, and mandatory mask use (Table 1).

**Table 1:** Brief descriptions of selected public health and social measures (PHSM) captured in the Health Intervention Tracking for COVID-19 (HIT-COVID) database.

Domain	Interventions	Details
Restrictions of travel and movement	Border closures	Control of population movement into the administrative unit
	Limiting movement within administrative unit borders	Closures of towns, cities, and roads, effectively limiting movement within an administrative unit
	Household confinement	Restricting individuals to their place of residence, including curfews, except when fulfilling essential needs
Social and physical distancing measures	Closures of public institutions and public areas	Closures of schools, offices, transportation, and public spaces such as parks
	Closures of non-public institutions and areas	Closures of office, leisure, entertainment, religious venues, restaurants, retail stores, nursing homes, and long-term care facilities
	Limiting gatherings	Limits on the size of social gatherings
Surveillance and response measures	Symptom screening at borders	Implementation of symptom screening upon arrival at an administrative unit
	Testing individuals	Testing individuals based on pre-specified symptom criteria for the presence of SARS-CoV-2 Testing individuals irrespective of symptoms
	Contact tracing	Tracing and monitoring contacts of identified cases
	Quarantine and home-isolation	Separating and restricting movements of individuals who may have been exposed (quarantine) or have symptoms and/or confirmed infection (isolation)
Other measures	Military and police deployment	Deployment of military and/or police to enforce COVID-19 related rules and restrictions
	State of emergency	State of emergency declarations which endow governments with additional powers to enforce policies
	Mandated face mask use	Requirements for the general population to wear a facemask in public

In order to collect these data, we recruited a global network of over 200 volunteer data contributors, primarily through professional and social networks associated with academic institutions of the management team. Upon agreeing to contribute to this project, volunteers were (1) assigned to follow and document policy changes for a specific country or administrative unit, (2) invited to attend an orientation meeting, which we held on a weekly basis for new recruits, (3) provided training materials including videos and written documentation on the expected workflow and nuances of data collection, (4) provided data entry templates and step-by-step instructions via emails, and (5) invited to an online social network for this project (www.slack.com) where they could ask and answer questions and interact with other members of the HIT-COVID team. This on-boarding process is summarized in our Data Entry Manual (Supplementary File 1).

**Fig. 1:**
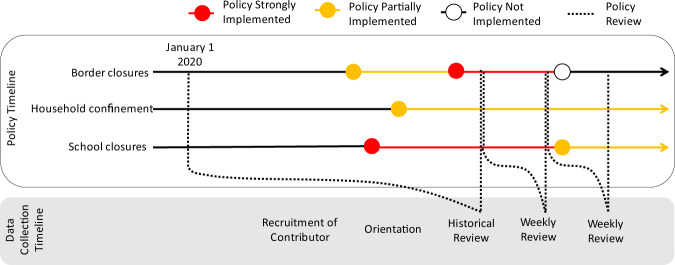
Schematic of data collection for an example administrative unit. Each point represents a date of policy change. Colors represent the degree or intensity of policy implementation. The time arrow goes from left to right. Dashed lines indicate the period over which data were collected.

We tracked the implementation of these interventions over time by asking contributors to submit an update each time a policy changed (Fig. 1). For each update, contributors collected the date of the update and the current status of the intervention, including whether the policy was recommended or required, and whether it was applied to the entire population or a subpopulation. In order to improve data quality and auditability, we also required an uploaded source document for each update (e.g., executive order, press release, or website screen capture).

We considered several sources of information for documenting policy changes, with a strong preference for official sources, including releases from national governments, ministries of public health, embassies and consular services. However, as these are not always available online, secondary sources such as media reports, technical and scientific reports were also deemed acceptable. Social media accounts (e.g., Facebook and Twitter) that are verifiably linked to national and subnational government entities (e.g., ministries of health) were also allowed if no better source could be found. When non-official sources were used, we asked contributors to corroborate information with more than one source where possible. In most cases, contributors were assigned to collect information based on their proficiency with the language(s) used in their particular administrative unit.

**Fig. 2:**
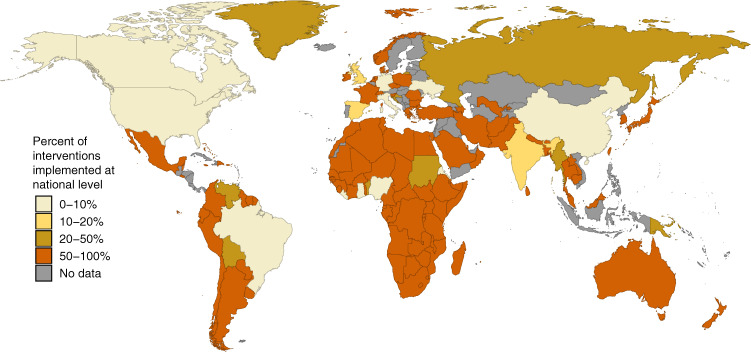
Spatial resolution of the data reported in each country. This figure shows the percentage of interventions reported at the national level for data reported from January 1, 2020 to July 10, 2020. As of July 10, there have been 10,129 records logged into our database covering 137 countries. A graduated color scale is used to show the percentage of national data available for each country, with the darkest shades representing the highest proportions of such data available. Countries in grey are those where no data have been recorded.

To ensure consistency and reproducibility, we developed a uniform data entry protocol (see Supplementary File 1, Data Entry Manual). First, by following a suggested search strategy, contributors were encouraged to conduct an overall review on public health intervention policies in their assigned geographic areas, which included national and first administrative units (Fig. 2). Second, contributors were asked to construct a historical timeline of all public health intervention policy changes starting from January 1, 2020, and complete historical data entry within one week. Third, contributors were asked to track their assigned locations and submit weekly updates either noting new policy changes (new policies or modifications of those previously implemented) or the absence of any changes (Fig. 1).

Data were entered into a survey designed on the Research Electronic Data Capture (REDCap) platform hosted at Johns Hopkins University^8^. If contributors discovered an error with their entries after submitting, they were asked to complete an online change request where they could log the details of the error. The management team reviewed all reported errors and updated the database as appropriate, reaching out to the contributor for clarifications via email or Slack when necessary. Further data validation was completed through weekly audit reports, which are described in detail in the Technical Validation section below.

As a living database, the HIT-COVID team modified the REDCap survey questions to capture nuances in re-opening policy as PHSMs have shifted from closings to openings over time. We disseminated standard information on what constitutes “open,” “partially closed,” and “fully closed,” and added questions about symptom screening and limits on opening capacity in different survey sections as appropriate. These changes are documented in the Data Entry Manual, and are also noted in the change log of our Github repository.

## Data Records

The latest version of data (V6.0 at the time of writing) and REDCap survey codebook are available on Github (https://github.com/HopkinsIDD/hit-covid) with the current release available through Zenodo (10.5281/zenodo.3939075)^9^. Data visualizations and a description of the project are available online at https://akuko.io/post/covid-intervention-tracking. In the database, each row represents a single policy update with a description of each of the fields below.

## Main Dataset

**unique_id:** unique id for the row combining the record_id and the intervention

**record_id**: unique id of the REDCap record (note that a single record is generated each time a set of data are entered, so these may be shared across interventions)

**entry_time:** time and date when data were entered by the contributor

**national entry:** flag for whether this is a national-level policy

**country:** ISO 3166-1 alpha-3 country code

**country_name:** country name

**admin1:** first administrative unit code (following GADM^10^ unless otherwise noted)

**admin1_name:** level 1 administrative unit name

**locality:** specified geographic areas below level 1

**usa_county:** name of county for USA county-level data

**usa_county_code:** FIPS code of the USA county

**intervention_group**: code that groups interventions by type

**intervention:** name of the specific intervention

**date_of_update:** date of updated status to policy implementation for a particular intervention

**status:** updated status of intervention policy

**status_simp:** simplified updated status of policy (partially implemented, strongly implemented, implementation suspended)

**subpopulation:** sub-population that the status of the specific intervention applies to

**required:** is the specific intervention required or recommended?

**enforcement:** are police/military enforcing the specific intervention?

**size:** what is the size of groups allowed for social gatherings or in restaurants?

**duration:** what is the duration of quarantine or self-isolation?

**testing_population:** sub-populations of symptomatic or asymptomatic populations tested

**details:** any specific details about the policy update

**source_document_url:** URL for the source document(s) stored in the online document repository

**url:** URL(s) provided by the contributors for the policy update

**entry_quality:** have these interventions been confirmed by the contributors (Verified, Changes pending, or Unverified)

### Completeness dataset

**country:** ISO 3166-1 alpha-3 country code

**admin1:** first administrative unit code (following GADM^10^ unless otherwise noted)

**usa_county_data:** does this completeness information refer to USA county-level data

**intervention_group**: code that groups interventions by type

**date**: date the contributor logged this completeness information

**completeness**: is this intervention information considered complete and up to date (Complete, Incomplete, Unsure)

## Technical Validation

Working with a large team of data collectors with diverse backgrounds presented unique challenges in assuring data quality. To continually clarify the intent behind the survey questions, we held online office hours twice per week to answer questions from contributors, and updated an online list of frequently asked questions that could be easily referenced by contributors. Contributors were encouraged to ask questions or raise concerns either through private correspondence or on our online social media community, within which contributors were organized into sub-communities by their assigned administrative units or themes (e.g., fragile states).

We employed a multi-faceted data validation process after data were reported. We emailed weekly audit reports to contributors for each sub-national administrative unit and country. These reports listed and visualized all intervention updates that had been logged up to that point, organized by intervention type. The reports also highlighted entries with potential errors including possible duplicated entries, potentially inaccurate dates (e.g. dates in the future), and field omissions. Contributors were asked to review their audit reports and either confirm that the entries were all correct, or log errors using an online form. The management team reviewed all logged errors and manually updated the information within REDCap. The study team maintains a record of all database changes, which can be made available upon request.

The database includes an “entry_quality” field that captures the results of this self-audit process. As a living database, this field will continue to change as more data are entered and validated, and as errors are found and resolved. All the records in an audit report for an administrative unit or country are considered “Verified” if the contributor who logged those entries (or in some circumstances someone else assigned to the same administrative unit) has confirmed that all of the information is correct or all reported errors have already been resolved by the management team. If the contributor logged an error for an audit report and the management team has not yet resolved the error, all entries associated with the report will state “Pending changes” in the “entry_quality” field. If a contributor has not submitted an audit report, these entries are considered “Unverified”.

When verifying their data through the audit reports, contributors were also asked to report whether the data for each intervention domain (Table 1) was “Complete”, “Incomplete”, or “Unsure”. Marking an intervention domain as “Complete” means either: 1) the policy is entered and up to date, 2) there is no policy, or 3) the level 1 administrative unit follows the national policy which is already captured in the database. This field provides information on whether the lack of data related to an intervention for a given spatial scale is likely due to the lack of a policy or simply missing data.

In addition to the weekly audits for contributor review, the management team continues to perform periodic internal audits to address repeated misunderstandings in the data entry process. Finally, on the public website visualizing these data (https://akuko.io/post/covid-intervention-tracking), we provide a form for viewers to flag potential errors which the management team will review, clarify, and change as necessary.

## Usage Notes

As a public database, HIT-COVID provides a unique opportunity for researchers and policy makers to unravel the potential impacts of PHSM on COVID-19 transmission worldwide. While there are many global databases collecting data for PHSM^3–7^, HIT-COVID’s particular strengths include the geographic scale of interventions captured across both national and first administrative units, a strong focus on underserved regions of the world (including Africa, South America, and South Asia, see Fig. 2), and standardized field definitions with items aimed at capturing the intensity and nuances of PHSM implementation and relaxation. Furthermore, HIT-COVID maintains a unique requirement for contributors to upload source documents to a centralized server. This archiving system will allow for accurate historical review of PHSM data that may otherwise be lost as government and media sources are updated. Finally, our multifaceted auditing process, based on continuous and scheduled exchanges between data contributors and the data management team, provides a way of verifying both the accuracy and completion of captured data. It is important for users of our data to ascertain whether the absence of data truly reflects the absence of policy implementation within the given PHSM category. HIT-COVID therefore represents a powerful complement to the strengths of other global databases, and has recently been aggregated to the WHO PHSM Database^7^.

**Fig. 3:**
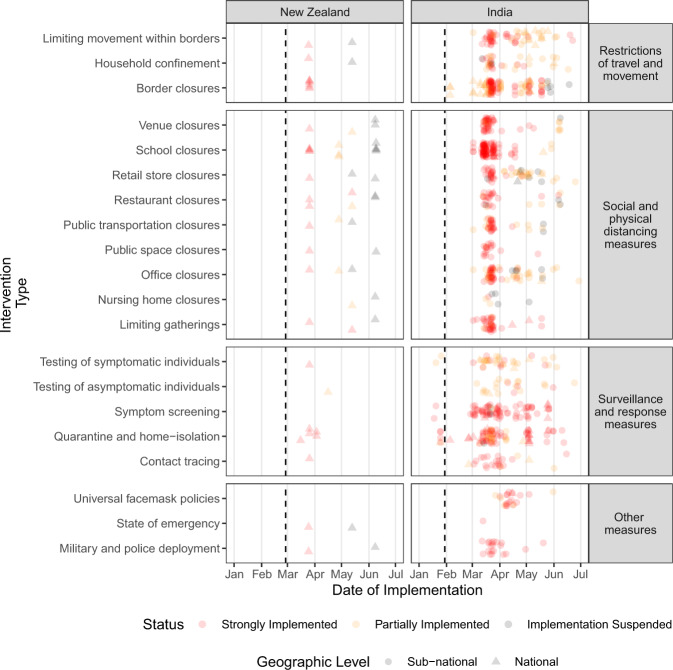
Illustrative example of implemented PHSM for data collected from New Zealand and India at the national and sub-national level to date. Each point represents an intervention. To illustrate interventions of the same type that occurred on the same date, the points are jittered vertically. Dashed line indicates when the first case of COVID-19 was reported in each country (February 28, 2020 and January 30, 2020, respectively).

Despite the global efforts made by all HIT-COVID contributors, there are still data missing from a number of countries (Fig. 2). Over the course of this pandemic, we will continue to address coverage gaps by recruiting more contributors, and are collaborating with other COVID-19 related health intervention databases (including the WHO PHSM database^7^). Furthermore, the scale and complexity of interventions that have been implemented are heterogeneous across regions. Although every effort was made to standardize intervention categories, there may be some discrepancies regarding how our pre-specified intervention categories were interpreted by contributors. While we continue to conduct internal audits to reconcile entries that may not have been coded correctly, further data validation and cleaning will be needed for country and region-specific analysis. Finally, as the pandemic progresses, we anticipate the need to modify or expand the fields of data collection in order to accurately reflect changes in the implementation of PHSMs, though this may make reconciliation of interventions over time more challenging. We provide an example of how our data may be eventually visualized to inform epidemiologic analyses (Fig. 3).

## Supplementary information

Supplementary File 1

## Data Availability

Codes for pulling the standardized dataset from HIT-COVID database and reproducing the figures on HIT-COVID website (https://akuko.io/post/covid-intervention-tracking) are available on Github repository (https://github.com/HopkinsIDD/hit-covid).
