# Supplementary material for: HIT-COVID, a global database tracking public health interventions to COVID-19
Source: Sci Data. 2020 Aug 27;7:286. doi: 10.1038/s41597-020-00610-2 (PMC7453020; doi:10.1038/s41597-020-00610-2)
Supplement: Supplementary file 1 [file 41597_2020_610_MOESM1_ESM.pdf]

Health Intervention Tracking of COVID-19 (HIT-COVID)

Data Entry Manual

Email hit-covid@jhu.edu if you have any questions or concerns.

Table of contents

Key Links 3

Background 3

General Overview 3

Key Types of Data 4

Data Sources 5

Spatial Scale/Administrative Unit of Data Entry 6

Entering and Updating Data 7
- Historical Data Entry 7
- Routine Data Entry 7
- Suggested Search Procedure 8

Data Field Definitions 9
- Mandatory 9
- Closed vs. Partially Closed vs. Open 9
- Subpopulations 10

Scope of Survey Domains 10
- Border Closures 10
- Limiting Movement within Administrative Unit Borders 10
- Household Confinement 11
- Quarantine and Home-Isolation 11
- Symptom Screening at Borders 11
- School Closures 11
- Nursing Home/Long-Term Care Closures 11
- Office Closures 11
- Leisure and Entertainment Venue Closures 11
- Religious Venue Closures 11
- Restaurant Closures and Restrictions 12
- Retail Store Closures 12
- Public Transportation Closures 12
- Public Space Closures 12
- Limiting Gatherings 12
- Contact Tracing 12
- Testing Symptomatic Individuals 12

Testing Asymptomatic Individuals 13
Universal Facemask Policies 13
Military and Police Deployment 13
State of Emergency 13
Other Interventions 13

Specific Examples of Partial Closures 14

Quality Assurance and Quality Control 15
Weekly Audit Reports 15
Fixing Incorrect Data in Previously Submitted Forms 15

Frequently Asked Questions 15

Contributor Expectations, Code of Conduct and Authorship 16
Code of Conduct 16
Authorship in Journal Publications 16

Key Links

Country contributor listing: this spreadsheet contains a list of countries and contact information for who is responsible for each
Code Book: The code book is also available on git here (in documentation directory)
Data entry video demo: this is an introduction on data entry
Online Data Entry: you can enter data here
Issue/Error Logging: any bugs or other issues with the survey should be logged this form
FAQs: you can check frequently asked questions here
Internal Dashboard: you can check record ids here
HIT-COVID website: the public website for HIT-COVID database
HIT-COVID Acknowledgement Form: We want to make sure you are acknowledged for your contributions to this project. If you don't see yourself on the team section of the website and would like to be, fill out this form.

Background

As the COVID-19 pandemic unfolds, massive government efforts are being made globally to try to reduce morbidity and mortality. Governments have taken a large range of actions, from broad-scale social distancing such as the forced lockdown of cities with mandatory home confinement, to behavior change campaigns to improve hand hygiene. Moreover, governments have implemented these measures at different points in time during the course of their epidemic.

Major government mandated actions come with huge economic risks and many are asking if some of the most drastic actions are worth it. In order to start to understand how different public health policy interventions may have influenced COVID-19 transmission across the globe, we need detailed data on when and where specific policy interventions have been enacted over the course of this epidemic. The goal of this project is to provide a comprehensive database of public health policy at the first level administrative unit, to serve as a key component of assessments of the impact of these policies on COVID transmission dynamics and other changes in the health of affected populations. This living database will be maintained throughout the course of the pandemic with visual summaries of raw data made available publicly.

General Overview

We will ask trusted contributors to provide data from reputable sources (typically government documents) on when specific policy changes occurred, specific groups the policy applies to and the degree to which these policies are recommended or required. Contributors will be tasked with providing updates on specific locations (countries or sub-national administrative units like provinces or states), matched to their language skills and interest, on a regular basis (e.g., every 2-3 days) including the original source documentation. At the start of this exercise contributors will be asked to do a review of the policy history since 1 Jan 2020 in each country entering key data through a series of online surveys. Every new change in policy should be logged with a new entry on REDCap. This includes opening, or changing the subpopulation that an intervention is applied (i.e. additional counties closing down or opening up).

Step 1: Historical Review: Contributors to conduct a historical review of interventions to construct a timeline of policy changes since 1-Jan-2020.
Step 2: Historical Data Entry: Contributors will enter data on the policy timeline through entering a series of surveys starting by entering the current policy state for each administrative unit.
Step 3: Regular Data Entry: Contributors will be asked to track policy changes in their assigned areas with updates (even those documenting no change in policy) at least once per week. If there are no intervention updates at the national and subnational levels this week, please log "no updates" in REDCap for each subnational unit you're covering.

Key Types of Data

We will focus on changes in policy in this exercise, not necessarily how well the interventions are adhered to. For all policies, we want to document the **dates** when they were implemented or changed (including both restrictions and relaxations of restrictions related to COVID-19). For most policies we also want to understand whether the policy was a recommendation or a requirement and whether this applies to the entire population or just to subpopulations (e.g., based on geography or demographics). The exact questions and codebook can be found here and the table below gives an overview. Further details can be found later in this guide.

**Domain**	**Questions**
Border Closures	• What restrictions have been placed on movement across borders (in and out)?<br>• How is this different for different modes of travels (air, land & sea)?
Symptom Screening	• Are travelers to the area supposed to be screened for symptoms?<br>• Is there a policy for symptom screening at checkpoints within the borders?
Quarantine and Self-Isolation	• For which populations have quarantine measures changed since the last update? Travelers, contacts of cases, other?<br>• For how long does quarantine last? Is it required or recommended?<br>• For which populations have home-isolation measures changed since the last update? Suspected cases, confirmed cases, discharged patients? Is it required or recommended?
Household Confinement	• Are people being asked to stay within their households (with the exception of essential trips)?<br>• Is this required or a recommendation?
Limiting Movement within Administrative Unit Borders	• What restrictions have been placed on movement within the borders of the administrative unit (closures of towns, cities etc or roadblocks)?<br>• Does this apply to all areas and subpopulations of the administrative unit, or just some?
School Closures	• Are there policies recommending or mandating the closure of schools?<br>• Does this differ between nursery, primary, and secondary schools?
Nursing Home/Long-term Care Closures	• What policies exist concerning visitors entering nursing homes & long-term care facilities?

Office Closures	● Are there policies recommending closure of offices and workplaces for non-essential workers?
Religious Venue Closures	● Are there policies recommending closure of religious venues?
Leisure and Entertainment Venue Closures	● Are there policies recommending closure of leisure and entertainment (e.g., movie theaters, night clubs, gyms)?
Restaurant Closures and Restrictions	● Are there policies recommending the closure of restaurants and bars in-person service?<br>● Are there policies limiting the maximum number of people allowed in a restaurant?
Retail Store Closures	● Are there policies requiring non-essential stores close?<br>● Essential stores typically include grocery stores, pharmacies, etc
Public Transportation Closures	● Are there policies closing or restricting public transportation?
Public Space Closures	● Are there policies closing or restricting public spaces?
Limiting Gatherings	● Are there limits on the number individuals that can gather?<br>● Are these mandatory? What is the maximum number allowed to gather?
Contact Tracing	● What policies exist to trace contacts of confirmed cases?<br>● Are quarantines imposed for the identified contacts?<br>● Does it depend on whether contacts are showing symptoms?
Testing Symptomatic Individuals	● Are symptomatic individuals eligible for testing according to policy?<br>● Does eligibility for testing depend on age, symptom severity, or other factors?
Testing Asymptomatic Individuals	● Are there policies in place that require or allow for testing of individuals regardless of symptoms?<br>● Does eligibility for testing depend on age, symptom severity, or other factors?
Mandated Face Masks	● Are there requirements for the general population to wear a facemask in public (not just symptomatic people)?
Military and Police Deployment	● Has the military and/or police been deployed to enforce COVID-19 related rules/restrictions?
State of Emergency	● Has a state of emergency been declared by the authorities of the area?

*The categories: mobile phone interventions, behavior change interventions, public funding of research, and economic and social safety net measures are optional and will not be included in weekly audits or public facing databases at this time.

Data Sources

- Given the fast pace of the pandemic, we will consider various sources of information in this exercise. As we are documenting policy, ideally all sources will be official government documentation but these may not always be available online. Secondary sources such as media reports, technical and scientific reports are also acceptable. Many ministries of health now post details on their social media (e.g., Facebook), which is considered an acceptable source of data should there be nothing more official.

- In order to properly check our data accuracy, part of our survey involves the submission of source documents. Specifically, data collectors should upload pdf documents of their sources. If multiple sources are used for a single domain, collectors should upload a zipped folder containing all pdf documents. Including a url is also helpful though websites may be edited or taken down over time.

Spatial Scale/Administrative Unit of Data Entry

- This project aims to document interventions at all **administrative level 1 units and national level** worldwide.
- In cases where there are national policy changes, no first level administrative unit should be selected. And you will be asked to confirm that the entry is for the entire country (See Figure 1 below).

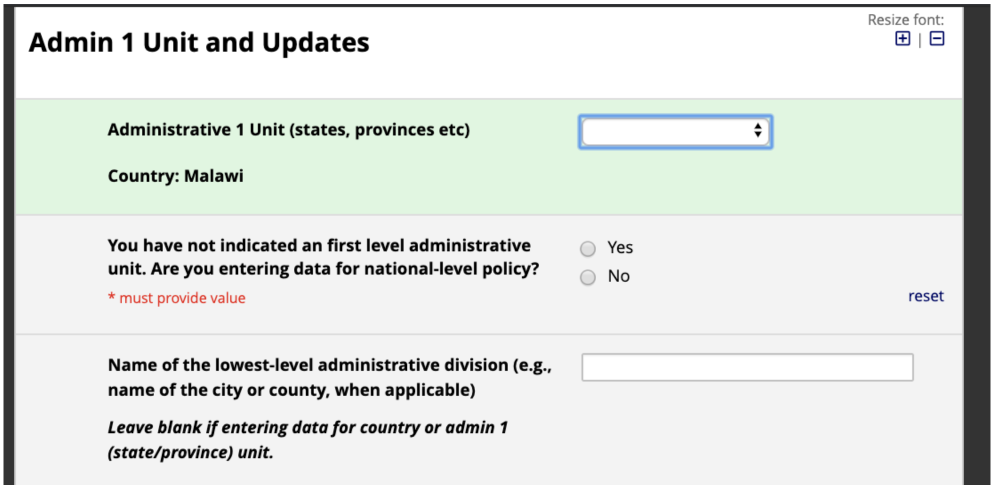

Figure 1: REDCap form to enter administrative-unit level data.

- For national-level interventions in countries with multiple people responsible for different admin 1 units, please coordinate national-level data entry between yourselves (See assignment spreadsheet for emails of others or in Slack).

Entering and Updating Data

After entering your name, email address and information on the geographic unit, you will be asked what domains of interest you have updates for (see below). If there have been no policy changes at the time of your report, it is still helpful to submit a survey with 'no updates' checked. There will be two types of data entry; [historical data entry](#) and [routine data entry](#), as described below.

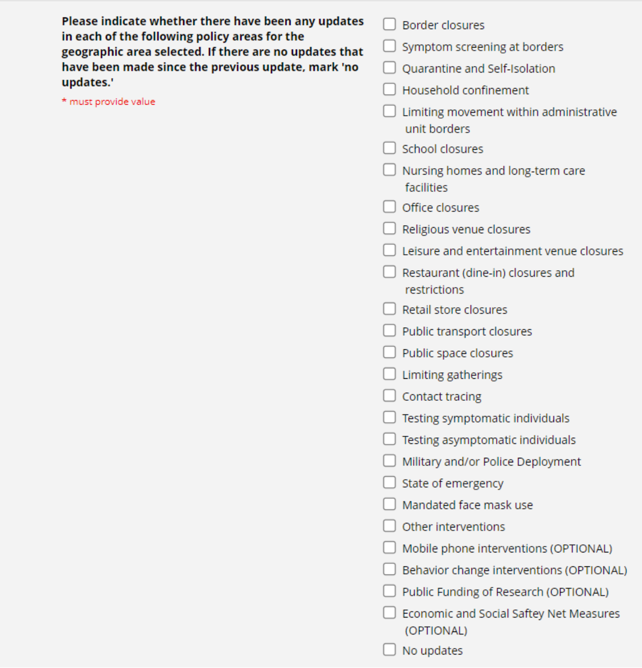

Please indicate whether there have been any updates in each of the following policy areas for the geographic area selected. If there are no updates that have been made since the previous update, mark 'no updates.'
* must provide value

- ☐ Border closures
- ☐ Symptom screening at borders
- ☐ Quarantine and Self-Isolation
- ☐ Household confinement
- ☐ Limiting movement within administrative unit borders
- ☐ School closures
- ☐ Nursing homes and long-term care facilities
- ☐ Office closures
- ☐ Religious venue closures
- ☐ Leisure and entertainment venue closures
- ☐ Restaurant (dine-in) closures and restrictions
- ☐ Retail store closures
- ☐ Public transport closures
- ☐ Public space closures
- ☐ Limiting gatherings
- ☐ Contact tracing
- ☐ Testing symptomatic individuals
- ☐ Testing asymptomatic individuals
- ☐ Military and/or Police Deployment
- ☐ State of emergency
- ☐ Mandated face mask use
- ☐ Other interventions
- ☐ Mobile phone interventions (OPTIONAL)
- ☐ Behavior change interventions (OPTIONAL)
- ☐ Public Funding of Research (OPTIONAL)
- ☐ Economic and Social Saftey Net Measures (OPTIONAL)
- ☐ No updates

Figure 2: Scope of interventions captured in HIT-COVID.

Historical Data Entry

- As many interventions started before data entry started, we ask that you do a review of the timelines of key interventions in each of your administrative units since 1-Jan-2020.
- Once you have a timeline of the policy changes, you can enter a series of updates that have no more than a single change within a domain with one submission (Figure 3).

Routine Data Entry

- As policies are changing rapidly, we ask that you try to monitor the situation in your assigned admin units and enter updates to specific domains as they are detected.
- **Even if no policy changes have been made, we ask that you file a report indicating no updates weekly**.
- If multiple policy changes occured since the last submission, you need to complete multiple surveys.
- **Every new change in policy should be logged with a new entry on REDCap.** This includes opening, or changing the subpopulation that an intervention is applied (i.e. additional counties closing down or opening up).

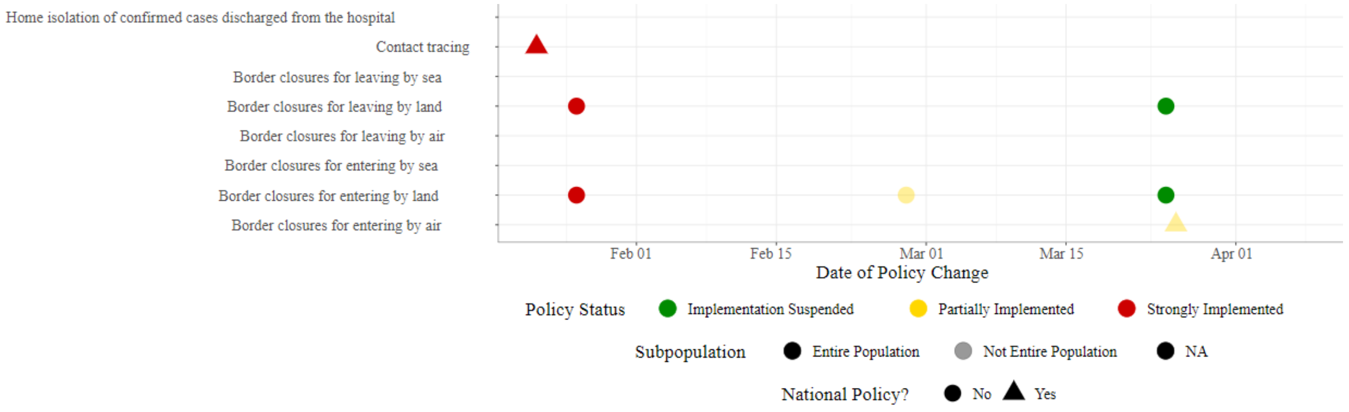

Figure 3: Intervention timelines as they appear on our internal dashboard.

Suggested Search Procedure

1. Please familiarize yourself with the breadth of policies that are listed in the table below.

2. The first task will be to **construct a historical timeline of all government interventions since January 1, 2020.** To begin, conduct a general search of policies related to your administrative unit. Some good places to start include:
    a. US Embassy websites usually give a good summary of the current situation in their related countries;
    b. Government websites;
    c. Health Ministries and/or Departments of Public Health websites;
    d. Social media (e.g. Facebook and Twitter) accounts linked to official state institutions and departments; and
    e. News report outlets.

3. As you come across different health interventions, please track them as you go along by entering data into the table found here. Enter the **date**, the **nature of the intervention** (this may be copied and pasted from official documents or decrees) and the **source URL.**

4. As you enter each health intervention within this table, make sure **you save a copy of the source URL** because you will be required to upload a file in the later data entry. This can be done by pressing “print” on your web-browser, and then saving the website as a PDF file.

    Ideally, all sources will be **official government documentation**, but we recognize that this is not always available. Secondary sources such as **newspaper reports, technical and scientific reports, and announcements made on social media** are acceptable if there is nothing more official.

    If more than one report pertains to a single intervention, please save these PDFs and/or screenshots into a zipped folder that can be uploaded at a later date.

5. Once you have finished filling out the table, please double-check your results with other publicly available databases to confirm your data, and to also fill in anything that may have been missed:

Organization	What it provides	URL

International Monetary Fund	Succinct overview of government measures in response to COVID-19.	https://www.imf.org/en/Topics/imf-and-covid19/Policy-Responses-to-COVID-19
Assessment Capacities Project (ACAPS) COVID-19 Government Measures	Aggregate overview of different interventions worldwide.	https://data.humdata.org/dataset/acaps-covid19-government-measures-dataset
Oxford COVID-19 Government Response Tracker	Aggregate overview of different interventions worldwide.	https://www.bsg.ox.ac.uk/research/research-projects/oxford-covid-19-government-response-tracker
Boston University Policy Tracking in the US	Policy tracking in the United States.	https://docs.google.com/spreadsheets/d/1zu9qEWI8PsOI_i8nI_S29HDGHIlp2lfVMsGxpQ5tvAQ/edit#gid=0
Institute for Health Metrics and Evaluation (IHME) Projections	Social distancing measures and dates across the United States of America.	https://covid19.healthdata.org/projections
National Conference of State Legislatures (NCSL)	Policy tracking in the United States	https://www.ncsl.org/research/health/state-action-on-coronavirus-covid-19.aspx
UNESCO School Closures	Use their interactive tool to determine when your country may have closed its schools.	https://en.unesco.org/covid19/educationresponse
International Civil Aviation Authority	Information on airport closures around the world.	https://www.icao.int/safety/Pages/COVID-19-Airport-Status.aspx
World Health Organization Public Health and Social Measures (PHSM) Database	Aggregated PHSM data from various databases (**including ours**)	https://www.who.int/emergencies/diseases/novel-coronavirus-2019/phsm

Data Field Definitions

Mandatory

- The government has mandated this activity. This does not mean it is necessarily enforced, but the policy has been made mandatory by the administrative unit.
- "Any part" of the policy being mandated means that at least some subset of the policy is mandated (e.g., closures for certain venue types or activities for certain populations)

Closed vs. Partially Closed vs. Open

- Closed means the venues are fully closed all of the time.
- Partially closed means there are some restrictions on the venue including:
  - Not all venue types in that category are closed
  - There are reduced hours for the venues
  - The venues are closed for some subpopulation(s) (such as demographic or geographic)
  - The venue capacity is reduced
  - For specific examples see the table later in this document

- Open means that there are no restrictions on the venue hours or capacity (there could still be requirements for maintaining distance, increased cleaning, mask wearing etc.)

Subpopulations

- Entire subpopulation - the intervention applies to essentially everyone in the admin1 unit or country
- Geographic subpopulation - the intervention applies to a geographic region within the admin1 unit or country (certain towns, districts, counties etc.)
- Demographic subpopulation - the intervention applies to a certain demographic subpopulation (such as those over a certain age, or those of a certain race/ethnicity)
- Other subpopulation - the intervention applies to some other subpopulation of the area (such as a certain type of worker, people of a certain religion, or anything that is not related to demographics or geography)

Symptom Screening

- These questions capture whether there are policies to implement symptom screening for people who go to a specific type of place/venue. If there is screening for anyone (employees, patrons, students, teachers etc.) at the venue then this would count as having symptom screening.
- If there is a symptom screening policy but no reduced capacity or reduced hours, the venue can be considered “open”.

Reduced Capacity

- This question captures whether there are policies which reduce the capacity of places/venues. This can include numbers of people or percentages of capacity.
- The reduced capacity limits should apply directly to the venues that are being logged (and not simply an extension of blanket gathering limits).
- If the policy simply states that people must maintain social distance, this does not count as reduced capacity.
- **If a venue has reduced capacity, it should be considered “partially closed”.**

Scope of Survey Domains

Border Closures

- This category captures policies closing borders to other countries or admin units
- Border policies are meant to be applicable to any admin unit but for many countries, these will be mostly policies around entering the country, not necessarily around borders between admin 1 units.
- This category is separated by type of transportation (land/sea/air) and whether there are restrictions on entering and/or leaving.

Limiting Movement within Administrative Unit Borders

- This category captures policies causing the closures of towns, cities etc and closures of roads. This is independent of mandates to stay at home or other advisories to maintain social distance.

Household Confinement

- This category captures policies that recommend or require people to stay at home with the exception of essential movements (e.g., grocery, doctors)

Quarantine and Home-Isolation

- Quarantine specifically refers to separating and restricting the movement of individuals who may have been exposed, but have not shown symptoms nor have been confirmed to have been infected
- Home-Isolation separates those who are symptomatic or have a confirmed infection.

Symptom Screening at Borders

- This category captures policies related to symptom screening in different populations including those people coming across borders or in specific checkpoints within borders.
- This does not include symptom screening within stores, workplaces, or schools which is captured within these individual domains.

School Closures

- We have indicated the categories of nursery, primary school, secondary school and post-secondary school. While school systems differ across the world, these are meant to correspond to <5 year olds (nursery), 5-11 year olds (primary), 12-18 year olds (secondary school) and >18 year olds (post-secondary).
- Universal school closures with the exception of very small groups like children of healthcare workers should be classified as 'full closure' with details of the exceptions provided in the free text.

Nursing Home/Long-Term Care Closures

- A long term care facility is: "facility-based long-term care services that include: board and care homes, assisted living facilities, nursing homes, and continuing care retirement communities" - NIA
- We are interested in the closure of these facilities to outside visitors (not the complete closure of the facilities themselves)

Office Closures

- This category captures policies to close offices and other workspaces that are not public facing venues such as leisure and entertainment and stores.
- Do not include essential businesses (e.g. supermarkets, pharmacies, healthcare) in this category.

Leisure and Entertainment Venue Closures

- This category captures policies closing large places for social gathering are closed for the purposes of social distancing.
- **Venues:** movie theaters and other types of theaters, casinos and nightclubs, golf courses, gyms and spas, private outdoor venues (pools, campgrounds, archery ranges, horseback riding, etc.)

Religious Venue Closures

- This category captures policies closing religious venues for services and other gatherings/events.
- This is a new category as of 6/2/2020, previously it was combined with leisure and entertainment venues.

Restaurant Closures and Restrictions

- This category captures policies closing dining establishments the purposes of social distancing and food handling safety.
- Restaurants are considered closed if they are only doing take-out and/or delivery
- **Venues:** restaurants, cafes, coffee shops, bars, food vendors

Retail Store Closures

- This category captures policies closing non-essential stores for the purposes of social distancing.
- **Essential stores typically include grocery stores or pharmacies while non-essential stores might include clothing retailers, malls, cosmetic stores, barber shops, salons, and bookstores**.
- Restrictions except for essential stores should be treated as fully closed.
  - If essential stores were regulated after/when the non-essential stores were closed, this update should be logged under retail store closures but keeping the status as fully closed mentioning the restrictions for essential stores in the details.
  - If in the rare case that only the essential work was regulated and non-essential work remained open, this should be updated under the “other” domain.
- **Venues:** bookstores, clothing stores, all other types of stores, hair salons, barber shops, nail salons, shopping malls, car washes, pet grooming, garbage cleaners (considered essential), postal services (considered essential)

Public Transportation Closures

- This category captures policies closing or limiting public transportation usage.

Public Space Closures

- This category captures policies closing public spaces including beaches and parks and the facilities at these locations.
- **Venues:** beaches, public parks and campgrounds, public pools, athletic fields, outdoor gardens, zoos

Limiting Gatherings

- This category captures policies to limit the size of gatherings to some number of people
- If there are different limits for indoor and outdoor gatherings, these should be logged as two entries with the details noting which type of gathering (indoor vs. outdoor)

Contact Tracing

- This category captures policies related to contact tracing describing how complete the process is.
- Complete contact tracing refers to the identification of all potential contacts, regardless of demographic factors. Partial contact tracing refers to the identification of some contacts who might be considered particularly at risk, e.g., finding household or elderly contacts of a confirmed case.

Testing Symptomatic Individuals

- This category captures policies related to who is eligible for testing **with** clinical symptoms.
- The categories of eligibility are: age, symptom severity, comorbidity, hospitalization, travel history, contact with case, other potential exposure, other

Testing Asymptomatic Individuals

- These questions are meant to capture policies related to who is eligible for testing **independent** of clinical symptoms.
- The categories of eligibility are: age (e.g., all people over 65), comorbidity, travel history, contact with case, other potential exposure, other

Universal Facemask Policies

- This is meant to capture policies that mandate the use of facemasks among people in the general public **without** symptoms during daily life (does not include health care workers).
- This includes policies recommending/requiring the public to wear masks when outside their homes, in all stores, etc. It does not include policies about mask use only in specific venues (i.e. health care settings and public transportation).

Military and Police Deployment

- This is meant to capture whether police, military or others have been specifically deployed to enforce COVID-related intervention policies.

State of Emergency

- This is meant to capture declarations of a state of emergency or state of disaster by the government.

Other Interventions

- Any interventions that are thought to be important that don't fit in the above categories can be logged under the "other interventions" section.
- Some common interventions logged here would be: prison closures, construction cessation, hotel closures, limiting access to non-essential health care.

Specific Examples of Partial Closures

Interventions	Partial Closure or Restriction - Examples
Border closures	Partially closed borders means they are closed to specific subpopulations, not that some border posts are closed and that others are opened (that would still be open)
Limiting movement within borders	Partially restricted refers to whether the policy applies to all within the admin unit or just some subpopulations
Household confinement	Curfews<br>Only those who are elderly or other high risk populations told to stay home<br>Only certain geographic areas are told to stay at home
School closures	Limited hours at school<br>Reduced number of days at school per week<br>Recommendations regarding online learning<br>Schools are only closed/restricted in certain geographic areas
Nursing home/long-term care closures	Limited visiting hours<br>Limits on number of day programs<br>Limits on acceptance of patients<br>These venues are only closed/restricted in certain geographic areas
Office (workplace) closures	Reduced working hours<br>Recommendations to work from home<br>Workplaces are only closed/restricted in certain geographic areas
Leisure and entertainment venue closures	Not all leisure and entertainment venues are closed<br>Reduced opening hours<br>Restrictions on number of patrons<br>Leisure and entertainment venues are only closed/restricted in certain geographic areas
Retail store closures	Restrictions on number of patrons<br>Some types of stores are allowed to open but not others<br>Retail stores are only closed/restricted in certain geographic areas
Religious venue closures	Limited opening hours<br>Restrictions on number of patrons<br>Religious venues are only closed/restricted in certain geographic areas
Public transportation closures	Reduced ticket sales<br>Reduced issuing of electronic transport passes<br>Reduced commuter volume<br>Only single or a few modes of transport affected (but not all)<br>Alternating access between subpopulations (e.g. males and females commute on separate days)<br>Public transportation are only closed/restricted in certain geographic areas
Restaurant closures	Reduced opening hours<br>Restrictions on number of patrons<br>Restaurants are only closed/restricted in certain geographic areas

* Note: venues which require mandated face-mask use, require people to maintain social distance and/or require symptom screening, but which are otherwise open, should be documented as fully “open”.

Quality Assurance and Quality Control

Weekly Audit Reports

- Each week, participants will be sent unverified audit reports detailing all entries for each country and admin unit they are responsible for in order to review that this information is correct. Bolded entries have not been verified.
- In the audits, an automated process is used to flag and highlight entries that **MAY** be errors. Please review these to make sure they are correct. If they are, no action is needed except for filling out the form to confirm that data looks OK. You will keep seeing them flagged each week but can ignore them. **Here are reasons data can be flagged:**
  - Date of update is more than 2 days after date of entry and the data is unverified
  - Date of update is more than 10 days after date of entry and data is unverified
  - Status is “unknown” (this option is no longer possible but was in early versions of the survey)
  - Potential duplicates (all information except the details are the same within 1 day)
  - The status is “open” before April 15th
  - The admin 1 unit is missing but the entry is not marked as a national entry
- As you look through your audits, please pay attention to highlighted intervention categories where the first entry is an intervention that is “open”. We want to emphasize that the first entries should reflect implementations of COVID-19 related policy. **If you come across a flagged entry where the first intervention logged is an “open”, please submit a change request for this to be altered or deleted.** The first entries under each intervention category should be either partial or full closure.
- ***It is very important that you verify the audits each week*** (or at least when the audits contain unverified entries) through the link internal to the audits. This is how your entries are marked as “Verified” in the public database.
- The management team will also perform occasional internal audits as it is deemed necessary. If you notice your information change from what you entered this is why.

Fixing Incorrect Data in Previously Submitted Forms

- If you make a mistake in a form that was submitted you can submit a correction using this form.

Frequently Asked Questions

- *For a more complete list of FAQs see this document.*
- *What if I select a domain to update but realize that it is not something I wanted to update?*
  In this case, you will be left with an incomplete survey in the survey queue once you are done updating all other domains. You can exit from here and your incomplete data from this domain will not be included in any cleaned datasets.
- *Should I include non-COVID-specific interventions?*
  Broad-scale policies occurring at country and admin 1 units that fall within one of the survey module categories should be included in the data collection. These are policies that may affect COVID-spread even if COVID is not specifically included in the policy description.
- *Which date should I enter when asked “When did this update occur?”?*
  Please enter the date that the policy is set to be implemented, not the date when the policy was announced.

- *Should I record negative intervention information (such as: no border closure or recommendations of not wearing masks) during the first update?*
  Since we're interested in policy changes and this is a continuation of no policy status, we don't need to record this kind of information **unless this is a change from a more strict policy.**
- *If border and public transportation closures apply to the entire population, with the exception of essential service workers (e.g. healthcare workers, military, and law enforcement), are these to be considered "partial" closure/restrictions or "closed"?* These should be considered **"closed"**, to reflect the idea that such closures apply to all but essential workers.
- *In my administrative region, there is a national decree to change the working hours to* x *number of hours, and only administrative services for the state are to remain open. How should this data be captured?* This would belong to the category of "**Office Closures**", and should be entered as a "partial" restriction.
- *What are some examples of "public spaces"?* We are defining "public spaces" as open recreation spaces like parks and beaches.
- *Where should I log closings and openings of barber shops and salons?* These should be logged under retail stores.
- *"One of the admin units didn't implement closures, but took away free public transport passes (usually given to seniors, students, etc) in an effort to reduce its use. Does that fall under this category (as "partial" closures, maybe)?"* This should be classified as a partial closure as it effectively reduces the volume of public transport riders.

Contributor Expectations, Code of Conduct and Authorship

Code of Conduct

The HIT-COVID team acknowledges the substantial time and effort contributors are putting into this project, particularly at a time of immense stress and uncertainty. In volunteering your time, we do ask you to meet a few basic expectations that will preserve the integrity of the data entered into this project:

1. The entered data is, to the best of your knowledge, accurate, complete, and up to date;
2. That, where possible, you participate in auditing the veracity of your entered data;
3. To communicate, via channels such as Slack and email, ongoing issues with regard to the interpretation of interventions and problems with online data entry; and
4. Where necessary, communicate with the HIT-COVID team should you need to scale down your volunteer commitments for any reason, including return to work and for personal reasons.

This code of conduct is ultimately non-binding, and we recognize that the success of this project rests in each volunteer playing their designated role to the best of their abilities. Please direct any concerns of questions to hit-covid@jhu.edu.

Authorship in Journal Publications

To honor your work, we invite all contributors to share in the authorship in an initial publication outlining the data collection process, which has been submitted to the journal *Scientific Data*. In following the model set by *The Lancet's* Global Burden of Disease (GBD) study, your authorship is expected to come in the form of acknowledgement as part of the **HIT-COVID Collaboration**, with your names indexed to PubMed and later appended at the conclusion of the manuscript. To meet the International Committee of Medical Journal Editors (ICMJE) guidelines for authorship, we ask you to read and approve of this publication (and any re-submissions), and fill out this form to offer your suggestions for improving the manuscript. As of June 2020, we are now in the

process of revising our manuscript, and prior to resubmission we will once again send the manuscript to seek your review.
